# Psychometric Validation of the Digital Competence Questionnaire for Nurses

**DOI:** 10.1177/23779608241272641

**Published:** 2024-10-01

**Authors:** Christoph Golz, Sabine Hahn, Sandra M.G. Zwakhalen

**Affiliations:** 1 School of Health Professions, Bern University of Applied Sciences, Bern, Switzerland; 2 Department of Health Services Research, 5211 Maastricht University, Maastricht, The Netherlands

**Keywords:** digital competence, nurse, questionnaire, validity, reliability

## Abstract

**Introduction:**

No brief questionnaire comprising knowledge, skills, and attitudes is available to measure digital competence among clinical practice nurses.

**Objective:**

The aim was to evaluate the structural validity and internal consistency of the Digital Competence Questionnaire (DCQ) for Clinical Practice Nurses.

**Methods:**

A cross-sectional study was conducted with English-speaking clinical practice nurses. Twenty-six items from an initial item pool, developed in a prior conducted Delphi Study, were included. Exploratory factor analysis for structural validity with “oblimin” rotation and a two-factor solution as well as internal consistency test using Cronbach's alpha were conducted.

**Results:**

Data from 185 nurses was obtained. The final questionnaire comprised of 12 items allocated to two factors: knowledge & skills and attitude. Factor “attitude” explained 33% of the variance and factor “knowledge & skills” 24%, resulting in a cumulative explanation of the variance of 57% by both factors. Internal consistency per factor was satisfactory, with 0.81 and 0.91, respectively.

**Conclusion:**

The DCQ for clinical practice nurses is valid and has satisfactory internal consistency. Researchers and nurse managers can use it to assess the level of digital competence among clinical practice nurses. Future psychometric validation of the DCQ for clinical practice nurses is required to allow a conclusion on the goodness of fit and reliability.

## Introduction

**Figure 1.**
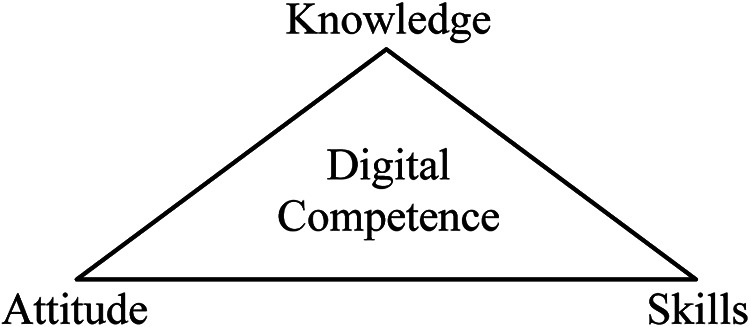
Framework of digital competence by Golz et al. (2023).

Digital competence is the degree to which an individual thinks he or she has the ability to interact with technology (Venkatesh & Bala, 2008). It comprises the theoretical understanding of how a technology can be used (knowledge), the ability to use the technology (skills), and the feelings toward technology or the way of behaving when interacting with technology (attitudes) (Kleib et al., 2021; Konttila et al., 2019) (Figure 1).

In times of digital progress in the health sector, it is becoming increasingly important for clinical practice nurses to have sufficient digital competence, since it has been found to be an inhibitor of technology-related stress at work (La Torre et al., 2019). The so-called technostress can lead to higher burnout symptoms or lower job satisfaction among nurses (Golz et al., 2021), which in turn are associated with increased intention to leave the organization (Peter et al., 2020). Clinical practice nurses spend most of their workday providing direct care to patients as face-to-face interactions in all settings within the health sector. They interact with technology, for example, by entering the information in the electronic health record and should be able to securely manage and transfer this data (Hübner et al., 2018).

### Review of Literature

Digital competence can be measured by using self-reported questionnaires. A scoping review from 2021 summarized fourteen questionnaires to measure nurses’ digital competence (Kleib et al., 2021). Most of these questionnaires focus on undergraduate or graduate students, nurse leaders, or nurse informaticists, and six of them were developed for nurses in clinical practice (Kleib et al., 2021). One of those is the Technology Informatics Guiding Educational Reform (TIGER)-Based Assessment of Nursing Informatics Competencies (TANIC), with 85 items comprising computer skills, information knowledge, and clinical information management (Hunter et al., 2015). Shortcomings of the available questionnaires measuring digital competence of clinical practice nurses include their sole focus on knowledge and skills (Kleib et al., 2021) and neglect of individuals’ attitudes at part of the digital competence definition (Konttila et al., 2019). A positive attitude toward technology at work in healthcare is associated with successful implementation and usage at work (Konttila et al., 2019). For example, it is known that technical issues and low reliability of implemented electronic health records lead to negative experiences among nurses with technology and consequently worsen their attitude towards using technology at work (Chang et al., 2016).

Most of the questionnaires summarized in the review of Kleib et al. (2021) such as the TANICS are lengthy and have more than 50 items and thus require much of the participants’ time to fill them out. The survey response rate has fallen sharply in recent years, and the forecast indicates a further decline (Stedman et al., 2019). Whereas in the 70s the response rate in surveys was high with approximately 75%, it is now usual to reach approximately 30%. The projected response rate for 2035 is expected to be near 20% (Stedman et al., 2019). There are different approaches to increase the response rate, such as the combination of different survey methods (e.g., phone, e-mail, and mail), but also the inclusion of the minimum questions needed to cover the questioned topics (Patten, 2016). Thus, to not burden nurses more than they are already through their work and still obtain adequate responses, research should aim to minimize the time needed to fill out questionnaires by obtaining their ability to measure the construct (Kleib et al., 2021).

Since no suitable questionnaire covers knowledge, ability and attitude, developing a short version based on existing questionnaires is impossible. Hence, a new brief questionnaire is needed to measure digital competence among nurses in clinical practice, paying attention to knowledge, skills, and attitudes. For this purpose, in a previous study, a Delphi Study was conducted, resulting in an initial item pool with 26 items with high content validity (average Content Validity Index = 0.95) (Golz et al., 2023). The aim of this study was, therefore, to evaluate the structural validity and internal consistency of the Digital Competence Questionnaire (DCQ) for clinical practice nurses.

## Methods

### Design

The development of the DCQ for clinical practice nurses was based on the Guidelines in Scale Development by DeVellis (2016). This includes the following eight steps: (a) Determine clearly what is to be measured, (b) generate an item pool, (c) determine the format of measurement, (d) have initial item pool reviewed by professionals with knowledge in the field, (e) consider inclusion of validation items, (f) distribute the survey, (g) perform item reduction, and (h) perform psychometric analysis of the reduced questionnaire. As preparation for the psychometric validation, an initial item pool with 26 items (knowledge *n* = 4, skills *n* = 8, attitude *n* = 14) was generated in the first five steps (Golz et al., 2023). In this paper, the focus was on the remaining three steps as described by DeVellis (2016): (f) survey distribution, (g) item reduction, and (h) psychometric analysis of the reduced questionnaire. The authors adhered to the STROBE (STrengthening the Reporting of OBservational studies in Epidemiology) checklist (see Supplementary file).

***Research questions:***

1. What is the factor structure of the Digital Competence Questionnaire (DCQ) for clinical practice nurses, and does it demonstrate adequate structural validity?
2. Does the Digital Competence Questionnaire (DCQ) for clinical practice nurses exhibit sufficient internal consistency across its subscales?

#### Sample

To test the construct validity and internal consistency of a questionnaire, 5–10 participants per item (question) of a scale are recommended (DeVellis, 2016). Therefore, the authors aimed for a sample size between 130 and 260 participants based on the initial item pool with 26 items. A combination of convenience and snowball sampling with English-speaking clinical practice nurses was conducted internationally to complete an online survey.

#### Inclusion/Exclusion criteria

The criteria for entering the study were a sufficient English language skills to understand the items and working as a clinical practice nurse. Participants did not have to be a native English speaker. Study participants self-assessed their English proficiency based on the introductory text and confirmed this by completing the survey. Regarding the profession, they were informed about the inclusion criteria in the preceding text, and in the survey the profession was asked. Persons who assigned themselves to other professions were excluded.

#### Data Collection

A cross-sectional study was conducted using the online survey tool SurveyMonkey^®^ between January and March 2022. Emails with information about the study's aim, inclusion criteria, data protection, and the survey link were sent directly to clinical practice nurses, and they were asked to forward the invitation to their colleagues. Private social media groups for clinical practice nurses on Facebook and Reddit were contacted and asked to forward the invitation for participation to their members. The study information and survey link were posted in private social media groups. Participation was voluntary.

#### Instrument

The instrument included questions on individual characteristics (age, country, and profession) and items related to digital competence. Only the question on profession was mandatory and designed to exclude participants from the analysis who did not belong to the sample. The 26 initial items on the DCQ for clinical practice nurses from the Delphi Study (Golz et al., 2023) were scored on a five-point Likert-Scale from 1 (“fully disagree”) to 5 (“fully agree”), with a high score indicating high self-perceived digital competence. The five-point Likert-Scale format was chosen because it is the most common item format for measuring opinions, beliefs, and attitudes (DeVellis, 2016).

#### Statistical Analyis

Data was analyzed using the statistical software R (R Core Team, 2021) and the package “psych.” Missing data was handled by listwise deletion if at least one item was missing from the 26 items on digital competence. The analysis comprised a descriptive analysis (mean, median and standard deviation, minimum, maximum, skewness, kurtosis) and exploratory factor analysis (EFA) for structural validity and an internal consistency test using Cronbach's alpha with satisfactory values >0.7 (Watkins, 2018). Skew and kurtosis were computed and evaluated for their potential impact on the EFA results with skewness ≥ ±2 and kurtosis ≥ ±7 (Watkins, 2018). The assumptions for an EFA were item correlations above 0.3, a significant Bartlett's test of sphericity, and Kaiser–Meyer–Olkin values ≥0.7 for the included items and all items combined (Watkins, 2018). If an item did not meet one of the criteria, it was excluded from the following steps, and all assumptions were re-evaluated. The rotation method “oblimin” was used for the EFA, as the included items were correlated (Watkins, 2018). The number of factors was chosen based on a scree plot and parallel analysis. Using the parallel analysis, the authors compared the random eigenvalues with the eigenvalues from the dataset. The number of factors was defined as the number of eigenvalues from the dataset exceeding the random eigenvalues. A factor was expected to comprise at least three items (Watkins, 2018). Cases with missing values for one item of the scale were excluded.

## Results

**Figure 2.**
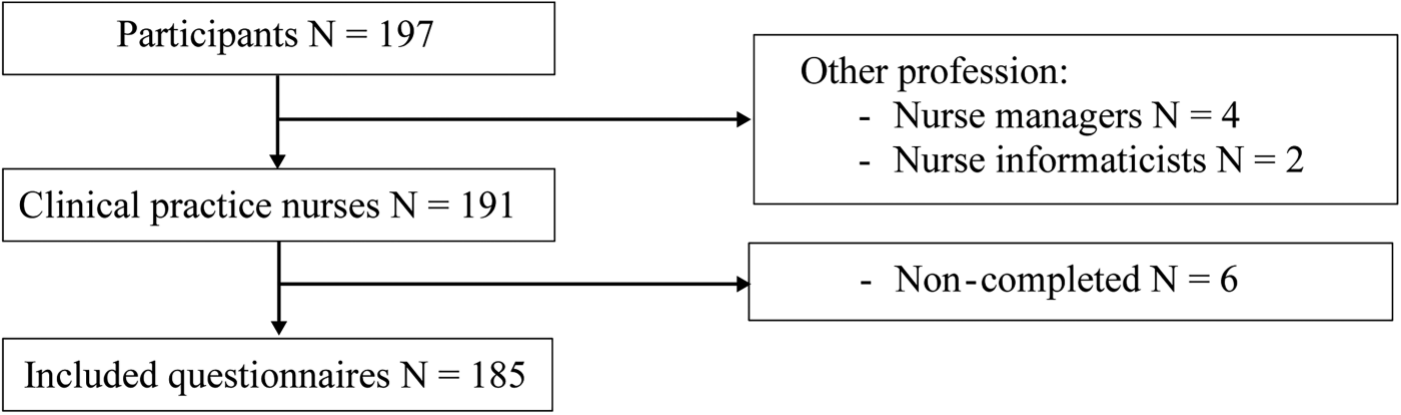
Flowchart detailing the number of included participants in the analysis.

Of the 197 individuals who responded to the online survey in March to April 2022, 6 reported having another function in nursing, such as nurse managers (*n* = 4) and nurse informaticists (*n* = 2), and were excluded from further analysis. Overall, 191 English-speaking clinical practice nurses participated in the study. Of these, 185 completed the questionnaire, resulting in inclusion of 93.9% of cases (Figure 2).

### Sample Characteristics

The mean age was 38.40 years (SD = 9.42). The majority was from United States of America (*n* = 39, 21%), followed by United Kingdom (*n* = 33, 18%), Australia (*n* = 25, 14%), Switzerland (*n* = 22, 12%), Canada (*n* = 17, 9%), Ghana (*n* = 12, 6%), Indonesia (*n* = 10, 5%), and other countries (*n* = 27, 15%). Most of the respondents were female (*n* = 134, 72%) and had a bachelor's (*n* = 81, 44%) or master's degree (*n* = 72, 39%) in nursing.

### Research Question Results

Table 1 summarizes the descriptive results of the 26 items. The median of the items ranged between 2 and 5, with high scores indicating a ceiling effect. Skew and kurtosis were not found to be above the cut-offs with <±2 for skewness and <±7 for kurtosis. On average, the participants took 6 min to complete the 26-item questionnaire.

**Table 1.** Descriptions of the Items.

Nr.	Topic	Item	Mean	SD	Median	Min	Max	Skew	Kurtosis
1	Knowledge	I am familiar with the digital technologies at my workplace.	4.34	0.76	4	1	5	−1.53	4.16
2	Knowledge	In general, I would rate my knowledge of digital technology as satisfactory.	4.23	0.76	4	2	5	−0.62	−0.34
3	Knowledge	I am familiar with the current laws and regulations pertaining to the protection and exchange of medical data (e.g., data protection, informed consent, and confidentiality) at my workplace.	3.96	0.92	4	1	5	−0.77	0.62
4	Knowledge	Patients use digital technologies to manage their symptoms themselves.	2.56	1.23	2	1	5	0.61	−0.56
5	Skills	I feel confident in dealing with confidentiality issues relating to digital technology at my workplace.	4.12	0.87	4	1	5	−0.58	−0.45
6	Skills	I feel confident about using digital technology to find relevant information.	4.44	0.69	5	3	5	−0.82	−0.56
7	Skills	I feel confident about using digital technology to share information.	4.24	0.77	4	2	5	−0.65	−0.41
8	Skills	I feel confident about using digital technology to communicate.	4.48	0.81	5	2	5	−1.63	2.07
9	Skills	I feel confident about the secure management of health data using digital technology.	3.91	1.08	4	1	5	−0.64	−0.51
10	Skills	I feel confident about using digital technology.	4.34	0.81	5	2	5	−0.93	−0.1
11	Skills	I feel confident about using digital technology to obtain data and information on clinical care.	4.44	0.62	5	3	5	−0.64	−0.57
12	Skills	I am able to reach conclusions based on information acquired through digital technologies.	4.23	0.77	4	2	5	−0.56	−0.67
13	Attitude	I am keen to use new digital technologies in my future professional practices.	4.46	0.74	5	3	5	−0.96	−0.56
14	Attitude	Digital technologies will make my day-to-day work easier.	4.21	0.85	4	1	5	−0.84	0.42
15	Attitude	I have an open attitude toward digital technology-related innovations at my workplace.	4.29	0.83	4	2	5	−0.98	0.22
16	Attitude	Digital technology fits well with the way I like to work.	4.21	0.87	4	2	5	−0.66	−0.78
17	Attitude	I enjoy using digital technology at my workplace.	4.20	0.87	4	2	5	−0.59	−0.94
18	Attitude	I encourage others to use digital technology in their professional practices.	4.26	0.82	4	2	5	−0.92	0.19
19	Attitude	I like to use digital technology at work.	4.25	0.88	5	2	5	−0.74	−0.72
20	Attitude	I am willing to improve my ability to use digital technology through further training.	4.39	0.76	5	2	5	−0.92	−0.17
21	Attitude	I believe that digital technology provides numerous benefits in terms of quality of care.	4.23	0.83	4	2	5	−0.79	−0.22
22	Attitude	I believe that digital technology improves clinical care.	4.26	0.81	4	2	5	−0.68	−0.64
23	Attitude	I believe that digital technology improves patient outcomes.	4.15	0.82	4	2	5	−0.56	−0.58
24	Attitude	I believe that digital technology is beneficial for my patients.	4.15	0.76	4	2	5	−0.61	−0.02
25	Attitude	I believe that digital technology is beneficial for health professionals.	4.37	0.76	5	2	5	−0.95	0.13
26	Attitude	I believe that digital technology is relevant for my future profession.	4.59	0.62	5	2	5	−1.52	2.36

### Structural Validity

Item 4 “Patients use digital technologies to manage their symptoms themselves” was excluded from subsequent steps due to an overall low correlation below the threshold of 0.3 (*r* = −.22 to −.26). The Bartlett's test for sphericity (*χ*^2^ (24) = 118.22, *P* < .001) with the remaining 25 items was significant and the KMO measure of sampling adequacy showed acceptable values above 0.7 (KMO = 0.83). The scree plot and parallel analysis proposed a three-factor solution. However, the third factor only comprised two items when conducting the EFA. Thus, the proceeded with a two-factor solution. Items 2, 7, 10, 13, 14, 15, 18, 20, 22, 25, and 26 were excluded stepwise from the analysis due to high cross-loadings above 0.4. Items 3 and 9 were excluded due to low factor loadings <0.4. The Bartlett's test and the KMO measure were re-evaluated for each iteration. The remaining item pool consisted of 12 items. The loadings per factor from the EFA with the 12 items are summarized in Table 2. For loadings above 0.4 the numbers are marked in bold. Factor 1 (Attitude) explained 33% of the variance and factor 2 (Knowledge & Skills) was 24%, resulting in a cumulative explanation of the variance of 57% by both factors.

**Table 2.** EFA Loadings.

	Attitude	Knowledge & skills
	Explained variance 33%	Explained variance 24%
Item	Factor 1	Factor 2
Digital technology fits well with the way I like to work.	**0** **.** **75**	−0.06
I enjoy using digital technology at my workplace.	**0** **.** **82**	0.01
I like to use digital technology at work.	**0** **.** **69**	0.08
I believe that digital technology provides numerous benefits in terms of quality of care.	**0** **.** **91**	−0.08
I believe that digital technology improves patient outcomes.	**0** **.** **72**	0.11
I believe that digital technology is beneficial for my patients.	**0** **.** **89**	−0.03
I am familiar with the digital technologies at my workplace.	0.11	**0** **.** **51**
I feel confident about using digital technology to find relevant information.	−0.07	**0** **.** **84**
I feel confident about using digital technology to communicate.	−0.03	**0** **.** **99**
I feel confident about using digital technology to obtain data and information on clinical care.	−0.1	**0** **.** **71**
I am able to reach conclusions based on information acquired through digital technologies.	0.12	**0** **.** **40**
I feel confident in dealing with confidentiality issues relating to digital technology at my workplace.	0.18	**0** **.** **45**

### Internal Consistency

All included items reached the conventional threshold of 0.7 for Cronbach's alpha, indicating sufficient internal consistency for the factor “Knowledge & Skills” with 0.81 (CI95% 0.79–0.82, *n* = 6 items) and for the factor “Attitude” with 0.91 (CI95% 0.90–0.93, *n* = 6 items). The exclusion of additional items resulted in a lower Cronbach’s alpha. Thus, the highest value was reached with the remaining 12 items.

## Discussion

This article demonstrates the structural validity and internal consistency of the 12-item DCQ for clinical practice nurses. The two factors explain a sufficient proportion of the variance, since it meets the average percentage of explained variance in behavioral science of approximately 57% (Peterson, 2000). The DCQ for clinical practice nurses can be completed in less than six minutes. The included items in the questionnaire were found to be relevant and sufficient by international panelists. They had an overall 37 items to rate and the possibility to add further essential items but ended with a 26-item pool (Golz et al., 2023). The reduction from the initial 26 items with a high content validity index to a 12-item questionnaire indicates that not all 26 items are needed to explain a satisfactory variation of the latent variables (DeVellis, 2016). This is confirmed by the high internal consistency of both factors included. Additional items would not lead to a higher explanation of variation and therefore can be considered redundant. The internal consistency of the factor knowledge and skills was above 0.9, which may be undesirable. Such a high value may indicate that the items within a factor measure the same phenomenon and are therefore unlikely to be a valid measure of the construct. As the values of the factors cluster around this threshold of 0.9 and are below 0.95, the authors consider the value acceptable (Hair et al., 2017).

Both factors fit the underlying theory of digital competence. Factor attitude (*n* = 6 items) assesses the participants’ attitude and factor knowledge and skills (*n *= 6 items) captures the knowledge and skills. Based on the gathered data, it was not possible to distinguish between knowledge and skills, although in theory they are two different entities of the concept “digital competence” (Le Deist & Winterton, 2005). This finding contradicts other studies evaluating a questionnaire for undergraduate nursing students, identifying separate factors for knowledge in informatics and informatics skills (Kleib et al., 2021). One reason might be that the initial item pool from the Delphi Study includes small numbers of items concerning knowledge (*n* = 4) and skills (*n* = 8). A factor should comprise at least three variables (Watkins, 2018), and with a starting point of four items for knowledge, this might have impeded the identification of knowledge as a separate factor. As a result, the individual factors of knowledge and skills are aggregated into one factor. Theoretically, this is not a problem, as knowledge and skills are related and explain a part of competence (Le Deist & Winterton, 2005). Furthermore, whereas attitude is a subjective feeling, belief or opinion, knowledge and skills have in common that they can be objectified and assessed by asking to describe, for example, available information systems (knowledge) or asking to save a file (skills) (Hunter et al., 2015). This commonality may make them more susceptible to loading on the same factor, as knowledge and skills ask for something factual and attitude involves a cognitive process considering other factors such as beliefs, feelings, and behavioral intentions toward technology. Nonetheless, since knowledge and skills are aggregated into one factor, a low score in the developed questionnaire does not allow determination of whether it is due to a shortage of knowledge or skills. The reasons for a low value in the factor knowledge and skills can be concluded based on the individual item scores. For a more in-depth examination of the identified low values and to determine the exact need for action, one may use existing comprehensive scales (Kleib et al., 2021). For example, the TANIC allows elaboration of whether the clinical practice nurse needs support updating data and information or communicating electronically with others, such as colleagues (Hunter et al., 2015).

Regarding the number of items in questionnaires measuring the digital competence of nurses, the DCQ is shorter (Kleib et al., 2021) without losing validity and reliability. For example, the reliability of other scales such as TANIC or the Canadian Nurse Informatics Competency Assessment Scale for measuring nursing informatics competence ranges between 0.81 and 0.99 (Kleib et al., 2021). Regarding validity, for some scales, only content validity was elaborated, or no validation was reported (Kleib et al., 2021). An exploratory factor analysis was also conducted for the Canadian Nurse Informatics Competency Assessment Scale, resulting in a scale with 21 items allocated into four factors, where a distinction could be made between knowledge and skills but without attitude (Kleib & Nagle, 2018a). 

### Strengths and Limitations

The development of this digital competence questionnaire, the DCQ, is based on the eight steps by DeVellis (2016), laying the basis for developing a theoretically sound and applicable questionnaire. Furthermore, it facilitates traceability of the process. The authors adhered to clear guidance regarding decisions based on the respective cut-off values, which has been mentioned as often missing in publications on factor analyses for scale development (DeVellis, 2016). Factor analysis is a robust method for identifying items that are performing better than others (DeVellis, 2016). To increase the method's robustness, the authors applied “oblimin” for rotation and used Spearman correlation due to the non-normal distribution of the data (Watkins, 2018). The planned minimum sample size was reached. However, the sampling method could have led to a sampling bias since technology-savvy clinical practice nurses might be more active on social media platforms. This could be one reason for the high ratings of self-perceived digital competence. Nevertheless, other studies also found high self-reported digital competence among nurses (Golz et al., 2021; Kleib & Nagle, 2018b; Kuek & Hakkennes, 2020), which might indicate that the sample is adequate for nursing. Furthermore, the mean age of 38 years for the participants does not suggest that only the digital natives of Generation Z have filled out the survey. Compared with the mean age of 43 years for the nursing population in the United Kingdom, for example, the age difference seems small (Skills for Care, 2022). Other studies show that older age is associated with lower digital competence among nurses (Golz et al., 2021; Kuek & Hakkennes, 2020). In this respect, the scale needs to be validated to see if it can differentiate between age groups. Despite the potential limitation through recruitment on social media platforms, social media as a recruitment platform gaining increasing interest and is a cost-effective solution to reaching a suitable sample of the target population (Welch, 2020). In this case, the use of social media recruitment expanded the reach of English-speaking participants.

Another reason for the high ratings of digital competence could be the self-perceived overestimation of incompetent individuals (Schlösser et al., 2013). The phenomenon is known as the Dunning-Kruger effect and describes individuals’ unawareness of their levels of competence. In particular, lower performing individuals were shown to overestimate their knowledge and skills (Maderick et al., 2016), which may apply to clinical practice nurses from countries with less digitalized health sectors. To avoid the problem of measurability, adjuvant objective tests are recommended, such as multiple-choice tests on, e.g., word processing (Maderick et al., 2016).

### Implications for Practice

The DCQ for clinical practice nurses still needs further psychometric testing. The current study misses, for example, information about reliability aspects like intra- and interrater reliability or test-retest reliability (DeVellis, 2016) and the sensitivity to change. Sensitivity to change is the ability of the questionnaire to identify actual differences between two measurements because of an intervention. This is especially informative when monitoring an intervention to improve the digital competences among nursing staff. Furthermore, other aspects of validity, such as discriminative validity, are needed to evaluate the questionnaire’s ability to discriminate between groups, such as technology-savvy vs. less technology-savvy clinical practice nurses. Further psychometric testing is also required using confirmatory factor analysis to confirm the underlying factor structure.

## Conclusions

The authors developed a short digital competence questionnaire for clinical practice nurses and added the dimension of attitude to the underlying competence construct. Researchers and practitioners can use the short questionnaire to elaborate on clinical practice nurses’ digital competence. Researchers could use the mean score as a primary outcome for intervention studies. Nurse managers may assess the level of digital competence at the entry of new clinical practice nurses or those already employed to identify needs. Clinical practice nurses can self-assess their digital competence to raise awareness of their competence and encourage reflection on further training in the use of technology at work. If needs are identified, an in-depth evaluation of the need for action is needed. Future psychometric validation of the DCQ for clinical practice nurses is required to allow a conclusion on the goodness of fit.

## Supplemental Material

sj-docx-1-son-10.1177_23779608241272641 - Supplemental material for Psychometric Validation of the Digital Competence Questionnaire for Nurses Supplemental material, sj-docx-1-son-10.1177_23779608241272641 for Psychometric Validation of the Digital Competence Questionnaire for Nurses by Christoph Golz, PhD, Sabine Hahn, PhD and Sandra M.G. Zwakhalen, PhD in SAGE Open Nursing

## Appendix

**Annex A:**

Number	Item
1	I am familiar with the digital technologies at my workplace.
2	In general, I would rate my knowledge of digital technology as satisfactory.
3	I am familiar with the current laws and regulations pertaining to the protection and exchange of medical data (e.g., data protection, informed consent, and confidentiality) at my workplace.
4	Patients use digital technologies to manage their symptoms themselves.
5	I feel confident in dealing with confidentiality issues relating to digital technology at my workplace.
6	I feel confident about using digital technology to find relevant information.
7	I feel confident about using digital technology to share information.
8	I feel confident about using digital technology to communicate.
9	I feel confident about the secure management of health data using digital technology.
10	I feel confident about using digital technology.
11	I feel confident about using digital technology to obtain data and information on clinical care.
12	I am able to reach conclusions based on information acquired through digital technologies.
13	I am keen to use new digital technologies in my future professional practices.
14	Digital technologies will make my day-to-day work easier.
15	I have an open attitude toward digital technology-related innovations at my workplace.
16	Digital technology fits well with the way I like to work.
17	I enjoy using digital technology at my workplace.
18	I encourage others to use digital technology in their professional practices.
19	I like to use digital technology at work.
20	I am willing to improve my ability to use digital technology through further training.
21	I believe that digital technology provides numerous benefits in terms of quality of care.
22	I believe that digital technology improves clinical care.
23	I believe that digital technology improves patient outcomes.
24	I believe that digital technology is beneficial for my patients.
25	I believe that digital technology is beneficial for health professionals.
26	I believe that digital technology is relevant for my future profession.
